# Evolution of Proteasome Regulators in Eukaryotes

**DOI:** 10.1093/gbe/evv068

**Published:** 2015-05-04

**Authors:** Philippe Fort, Andrey V. Kajava, Fredéric Delsuc, Olivier Coux

**Affiliations:** ^1^CNRS, CRBM, UMR5237, Montpellier, France; ^2^Université de Montpellier, France; ^3^Institut de Biologie Computationnelle, Montpellier, France; ^4^CNRS, IRD, Institut des Sciences de l’Evolution, UMR 5554, Montpellier, France

**Keywords:** proteasome, PA700, PA200, PA28, PI31, evolution

## Abstract

All living organisms require protein degradation to terminate biological processes and remove damaged proteins. One such machine is the 20S proteasome, a specialized barrel-shaped and compartmentalized multicatalytic protease. The activity of the 20S proteasome generally requires the binding of regulators/proteasome activators (PAs), which control the entrance of substrates. These include the PA700 (19S complex), which assembles with the 20S and forms the 26S proteasome and allows the efficient degradation of proteins usually labeled by ubiquitin tags, PA200 and PA28, which are involved in proteolysis through ubiquitin-independent mechanisms and PI31, which was initially identified as a 20S inhibitor in vitro. Unlike 20S proteasome, shown to be present in all Eukaryotes and Archaea, the evolutionary history of PAs remained fragmentary. Here, we made a comprehensive survey and phylogenetic analyses of the four types of regulators in 17 clades covering most of the eukaryotic supergroups. We found remarkable conservation of each PA700 subunit in all eukaryotes, indicating that the current complex PA700 structure was already set up in the last eukaryotic common ancestor (LECA). Also present in LECA, PA200, PA28, and PI31 showed a more contrasted evolutionary picture, because many lineages have subsequently lost one or two of them. The paramount conservation of PA700 composition in all eukaryotes and the dynamic evolution of PA200, PA28, and PI31 are discussed in the light of current knowledge on their physiological roles.

## Introduction

Maintenance of cellular proteostasis is a crucial challenge for all living organisms. Cells must selectively degrade proteins in a timely manner to control their individual level, to eliminate active proteins and thus terminate biological processes and to remove altered proteins in order to prevent their deleterious effects or accumulation. Proteasomes are high molecular weight cellular complexes that degrade cytosolic and nuclear proteins into peptides in eukaryotic cells (Goldberg 2007). Proteasomes are responsible for most of nonlysosomal proteolysis. The catalytic core of proteasomes, termed the 20S proteasome or the core particle, is a barrel-shaped assembly of four stacked rings. All 20S proteasome subunits are related and can be classified in two families, called α and β (Coux et al. 1994). The two inner rings are identical and consist of seven different β subunits, whereas the two outer rings, also identical, consist of seven α subunits. Among the seven β subunits, three (β1, β2, and β5) bear catalytic sites responsible for the three peptidase activities of the complex (i.e., chymotrypsin-like (β5), trypsin-like (β2), and caspase-like (or postglutamyl peptide hydrolysis, PGPH) (β1)). These catalytic sites are enclosed into the chamber formed by the two β rings. Access of substrates to the chamber is controlled by the α rings that form on each side a pore. The opening of the pores is itself controlled by a “gate” made by the N-terminal ends of the α subunits; this gate is usually closed and is opened upon binding of activating proteins (proteasome activators, PAs) to the α rings. PAs are thus critical components in proteasome-dependent proteolysis. To date, three types of PAs have been identified: PA700 (also called 19S complex; Chu-Ping et al. 1994), PA200 (Ustrell et al. 2002), and the PA28 complexes (also called 11S regulator or REG; Dubiel et al. 1992; Ma et al. 1992). Another proteasome regulator, PI31, has been identified (Chu-Ping et al. 1992). Contrary to PAs that activate 20S proteasome in vitro, PI31 can inhibit proteasome peptidase activities but its real contribution to proteasome functions is still a matter of debate (Bader et al. 2011; Li et al. 2014). PA700 binds to the 20S proteasome in an ATP-dependent manner and the resulting 26S proteasome has a major role in the control of cell homeostasis by degrading proteins labeled by ubiquitin tags as well as certain nonubiquitylated proteins. In contrast, PA200, PA28, and PI31 do not require ATP to bind to the 20S and the resulting complexes target ubiquitin-independent protein degradation, unless they are part of a hybrid form of the proteasome that contains PA700 on the other side (Hendil et al. 1998; Tanahashi et al. 2000).

20S proteasomes have been identified in eukaryotes, archaea, and in bacteria of the Actinomycetes phylum (Gille et al. 2003). Most of bacteria that do not code for a 20S proteasome homolog express another proteolytic structure made of a homododecamer of the ClpQ/HslV protein associated with one or two hexamer(s) of the ClpY/HslU ATPase (Rohrwild et al. 1996). The ClpQ/HslV complex is considered as the phylogenetic ancestor of the 20S proteasome (Bochtler et al. 1999).

The 20S α and β subunits share structural similarity and likely originated from an ancestral gene that duplicated before the divergence of archaea and eukaryotes (Gille et al. 2003). In contrast to the 20S proteasome, the evolutionary history of PAs remains fragmentary and scattered. Here, we present a comprehensive view of the evolution of the three types of activators and of PI31 from archaeal to eukaryotic lineages, using the classification of eukaryotes recently revised by Adl et al. (2012). We examined genomic data available for a total of 17 clades, spreading over 3.5 billion years of evolution and covering archaea and most of the eukaryote supergroups, that is, Opisthokonta (including Metazoans, Choanoflagellida, Ichthyosporea, and Fungi), Amoebozoans, Excavates (including Metamonads [Diplomonadida and Parabasalia] and Discoba [Heterolobosea and Englenozoa/Kinetoplastids]), Archaeplastida (Choloroplastida and Rhodophyceae), SAR (Stramenopiles, Alveolates, and Rhizaria), and two unclassified clades, Cryptophyta and Haptophyta, previously classified as Chromalveolates with the SAR group. We show that the full current repertoire of proteasome regulators was already present in the last eukaryotic common ancestor (LECA) and has subsequently evolved through independent duplication/loss events in specific lineages.

## Materials and Methods

### Genomes

Most of sequences were retrieved from NCBI annotated database (nr and EST, http://www.ncbi.nlm.nih.gov), using NCBI PHI-BLAST as well as BLAST and Annotation search tools available in the Geneious 7.1.5 package (Biomatters, http://www.geneious.com/). For specific searches, additional genome browsers were used as follows: Vertebrate and chordate genomes were searched using keyword or BLAST/BLAT search tools available in Ensembl (http://www.ensembl.org/, [Flicek et al. 2014]). For Testudines and Archosauria, genomes of 48 bird species as well as genomes of alligator (*Alligator mississippiensis)* and green turtle (*Chelonia mydas*) were searched at http://phybirds.genomics.org.cn/index.jsp. Cartilaginous fish elephant shark (*Callorhinchus milii*) was searched at http://esharkgenome.imcb.a-star.edu.sg/, and lamprey (*Petromyzon marinus*) at http://jlampreygenome.imcb.a-star.edu.sg/. Lancelet (*Branchiostoma floridae*) and sea urchin (*Strongylocentrotus purpuratus*) were searched on the UCSC Genome Browser (http://genome-euro.ucsc.edu). Genomes of Hemichordates (*Saccoglossus kowalevskii*), Cnidaria (*Hydra magnipapillata*, *Nematostella vectensis*), Choanoflagellates (*Monosiga brevicollis*), and Placozoa (*Trichoplax adhaerens*) were searched at http://www.metazome.net/. Apusomonadida genome sequences (*Thecomonas trahens*) were searched at http://www.broadinstitute.org/annotation/genome/multicellularity_project/. Protist data were searched at JGI (http://genome.jgi-psf.org/), in particular Cryptophyta (*Guillardia theta*), Haptophyta (*Emiliana huxleyii*), Heterolobosea (*Naegleria gruberi*), and Stramenopiles (*Thalassiosira pseudonana*, *Phytophtora ramorum*). Pathogenic protists were specifically searched on EuPathDB (http://www.eupathdb.org/eupathdb/), gathering data of many species from Alveolates (*Plasmodium*, *Cryptosporidium*, *Toxoplasma*, *Theileria*, and *Babesia*), Amoebozoa (*Entamoeba*), Diplomonadida (*Giardia*), Euglenozoa (*Trypanosoma*, *Leishmania*), Fungi (*Encephalitozoon*), and Parabasalia (*Trichomonas*). Dynophyceae data (*Symbiodinium* sp. clade B1) were analyzed at http://marinegenomics.oist.jp/. Transcriptomes of the dipters *Episyrphus balteatus*, *Megaselia abdita* (Brachycera Cyclorrhapha Aschiza), and *Clogmia albipunctata* (Nematocera Psychodomorpha) were analyzed at http://diptex.crg.es/. (All URLs were last accessed on April 28, 2015.)

### Sequence Alignments

Amino acid sequences were aligned using MAFFT v7.017 (Katoh et al. 2002) or MUSCLE (Edgar 2004) programs, available in the Geneious 7.1.5 package (Biomatters, http://www.geneious.com/, last accessed April 28, 2015). Multiple sequence alignments (MSA) were manually edited and processed by Gblocks at http://molevol.cmima.csic.es/castresana/Gblocks_server.html (last accessed April 28, 2015) (Talavera and Castresana 2007) to remove poorly aligned and divergent regions, except for PA28 for which BMGE (block mapping and gathering with entropy; Criscuolo and Gribaldo 2010) with a 0.6 cut-off value was used instead. Nucleotide MSAs were performed using the translation align tool implemented in Geneious. For the detection of the arrays of HEAT-like repeats in PA200 sequences, a sequence profile method (Bucher et al. 1996) was used as described (Kajava et al. 2004). For more detailed comparison of the HEAT-like repeat arrays, the MSA of PA200 proteins was generated by using the sequence profile (Bucher et al. 1996) built from the alignment of several PA200s that are the most similar to the *Saccharomyces cerevisiae* protein of known 3D structure.

### Phylogenetic Analyses

Phylogenetic trees were estimated both by maximum likelihood (ML) (PhyML; Guindon and Gascuel 2003) and Bayesian approaches (MrBayes; Ronquist et al. 2012), as implemented in Geneious. Both are probabilistic methods based on the likelihood function. ML returns the topology that maximizes the likelihood function and computes nonparametric bootstrap percentages to estimate node support (i.e., robustness of the topology). The Bayesian approach samples trees according to their posterior probability (PP) and directly estimates clade PPs as a measure of node support. Best-fitting models for amino acid and nucleic acid substitution were chosen using ProtTest (Abascal et al. 2005) and jModelTest (Darriba et al. 2012), respectively. In most of amino acid MSAs, the best-fitting model was LG + I + G. PhyML was set-up using the gamma shape and proportion of invariable site parameters produced by ProtTest. ML trees were optimized for topology, length and rate and were generated using the best of nearest-neighbor interchange and subtree-pruning-regrafting tree search algorithms, with 200 bootstrap replicates.

**Fig. 1.—:**
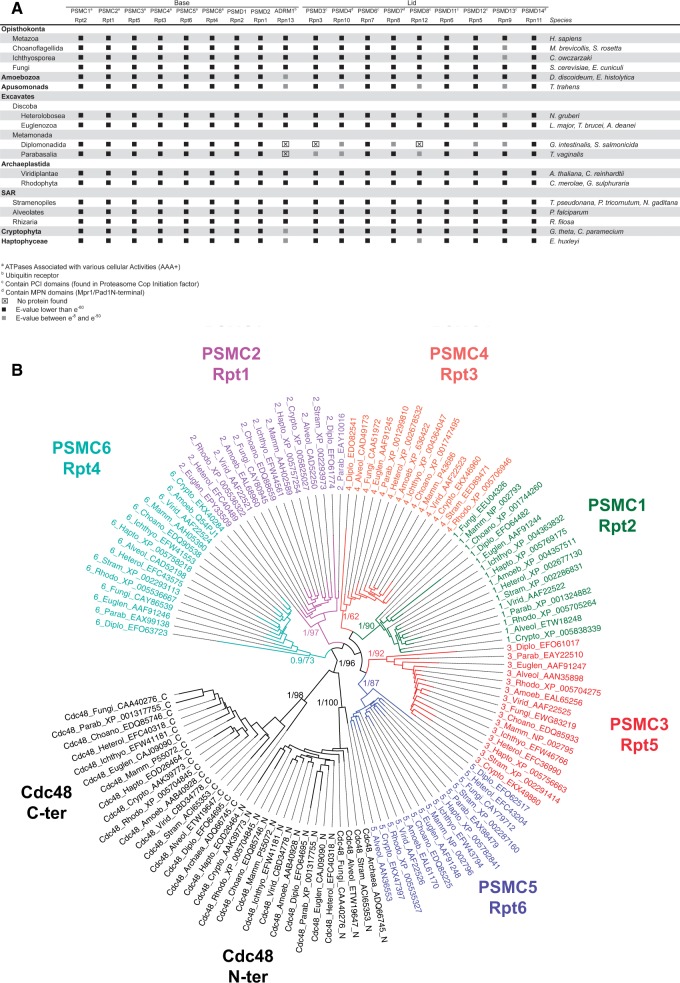
High conservation of PA700 subunits in the eukaryote supergroups. (*A*) Synopsis of PA700 subunits distribution in major supergroups. Sequences were identified by reciprocal Blast searches. Black squares: E-values lower than e^−80^, gray squares: E-values between e^−8^ and e^−80^. Taxonomy is indicated on the left and the corresponding species on the right. (*B*) Phylogenetic tree of the six AAA+ ATPases subunits. The ATPase domains were aligned and trees were produced by PhyML and MrBayes (see Materials and Methods). The two ATPase domains of Cdc48 were used as outgroups. Only PPs and bootstrap proportions of relevant nodes are indicated.

**Fig. 2.—:**
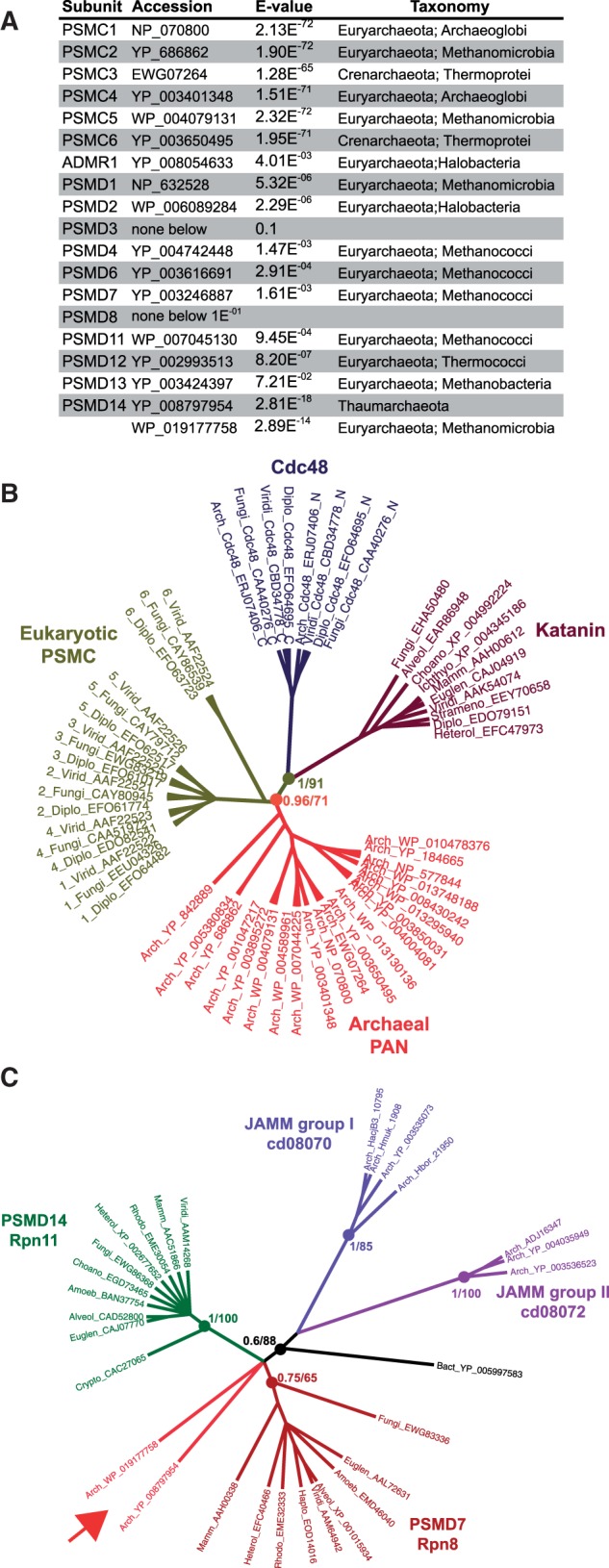
Archaeal orthologs of PSMC and PSMD7/PSMD14. (*A*) Similarity of archaeal proteins with PA700 subunits. Full-length sequences of eukaryotic PA700 subunits were used as queries against archaeal data. Indicated are the accession numbers, the E-values and the taxonomic status of the best hits. Only PSMCs and PSMD14 produced significant scores. (*B*) Archaeal orthologs of PSMCs. Trees were produced from eukaryotic and archaeal ATPase domains MSA by PhyML and MrBayes (see Materials and Methods). The two ATPase domains of Cdc48 (_N and _C) and Katanin were used as subfamily outgroups (Iyer et al. 2004). (*C*) Archaeal orthologs of PSMD7/PSMD14 (arrow). Archaea JAMM groups were defined by Hepowit et al. (2012). Trees were produced from eukaryotic and archaeal MPN domains MSA by PhyML and MrBayes (see Materials and Methods). Filled circles figure nodes critical for orthology. Adjacent numbers indicate PPs and bootstrap proportions.

**Fig. 3.—:**
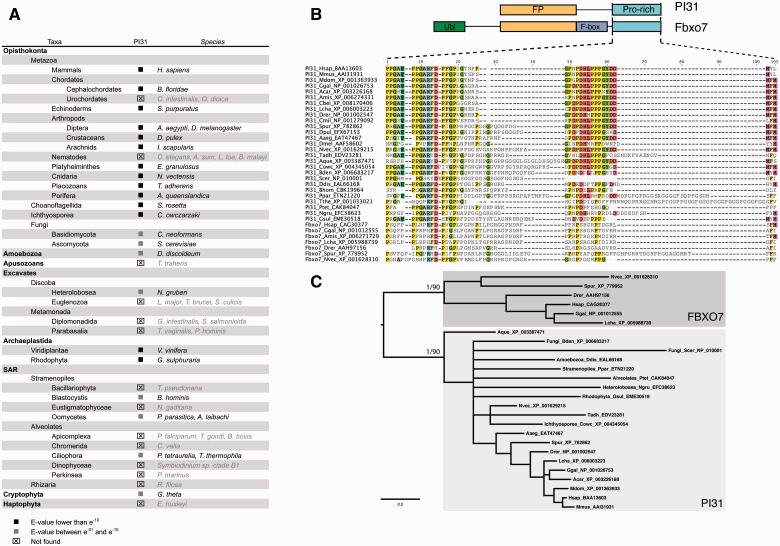
PI31/PSMF1 distribution and losses in eukaryotic supergroups. (*A*) Synopsis of PI31 distribution in major supergroups. Sequences were identified by reciprocal Blast searches. Black squares: E-values lower than e^−10^, gray squares: E-values between e^−1^ and e^−10^. ⊠: no homologous sequences found. Taxonomy is indicated on the left and the corresponding species on the right. Names of species missing PI31 are grayed. (*B*) PI31 and its interactor Fbxo7 share the FP (Fbxo7 and PI31) domain and a proline-rich domain (upper panel). Fbxo7 also includes an ubiquitin-like domain and an F-box domain. Multiple protein sequence alignment of the proline-rich domain show differences between PI31 and Fbxo7, in particular in the central motif. (*C*) Phylogenetic tree of PI31 and Fbxo7. PI31 and Fbxo7 sequences were aligned and trees were produced by PhyML and MrBayes (see Materials and Methods). Only PPs and bootstrap proportions of relevant nodes are indicated.

**Fig. 4.—:**
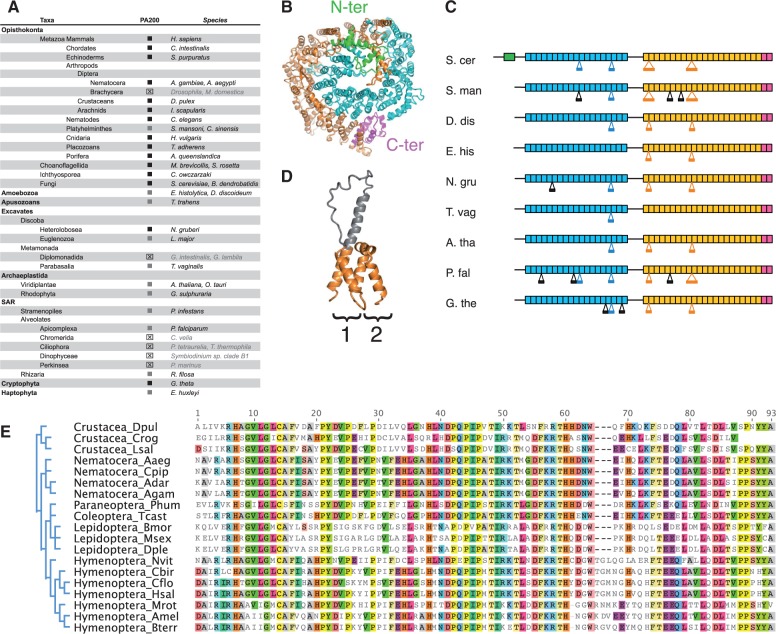
PA200 distribution and losses in eukaryotic supergroups. (*A*). Synopsis of PA200 distribution in major supergroups. Sequences were identified by reciprocal Blast searches. Black squares: E-values lower than e^−80^, gray squares: E-values between e^−8^ and e^−80^. ⊠: homologous sequences not found. Taxonomy is indicated on the left and the corresponding species on the right. Names of species missing PA200 are grayed. (*B*) Crystal structure of proteasome activator Blm10/PA200 from *Saccharomyces cerevisiae* (S. cer) (Sadre-Bazzaz et al. 2010) when viewed from the top of the complex with the 20S proteasome (which is omitted for the sake of the clearness). The structure represents a long curved α-solenoid folded on itself. The Blm10/PA200 crystal structure lacks two unstructured regions that link the N-terminal (green), the first α-helical (blue) and the second α-helical one (yellow), ended by the conserved C-terminal Pfam PF11919 domain (magenta). (*C*) Schematic representation of Blm10/PA200 proteins from different organisms. The upper S. cer protein has the known 3D structure while the others were deduced based on the sequence similarities with the S.cer protein. Rectangles denote α-solenoid structures with HEAT repeats. The color code is the same as on panel B. Black lines connecting the rectangles show regions that were not resolved by the X-ray crystallography. Large insertions of more than 40 residues into the core of the α-solenoids are shown below the rectangular boxes. The insertions that are observed in the 3D structure are colored, while ones that are predicted based on the sequence alignment are in black. The predicted insertions may have structures as shown on panel (*D*). S. man, *Schisostoma mansoni* (Platyhelminthes); D. dis, *Dictyostelium discoideum* (Amoebozoa); E. his, *Entamoeba histolytica* (Amoebozoa); N. gru, *Naegleria gruberi* (Heterolobosea); T. vag, *Trichomonas vaginalis* (Parabasalia); A. tha, *Arabidopsis thaliana* (Viridiplantae); P. fal, *Plasmodium falciparum* (Alveolates); G.the, *Guillardia theta* (Cryptophyta). (*D*) An example of a large insertion (gray color) into the HEAT repeat unit (1) in comparison with a typical HEAT repeat unit (2). (*E*) Absence of PA200 in Brachycera insects. Alignment of PA200 orthologs showed that the C-terminus is highly conserved among arthropods and if present, should have been detected in Brachycera.

**Fig. 5.—:**
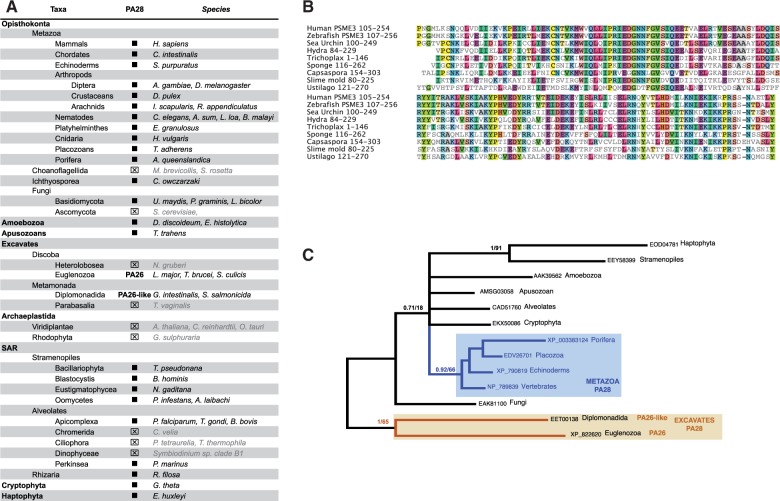
PA28 distribution and losses in eukaryote subgroups. (*A*) Synopsis of PA28 distribution in major supergroups. Sequences were identified by reciprocal Blast searches. Black squares: E-values lower than e^−80^. ⊠: homologous sequences not found. Taxonomy is indicated on the left and the corresponding species on the right. Names of species missing PA200 are grayed. The putative ortholog of the Trypanosoma PA26 is indicated as PA26-like. (*B*) Sequence alignment of PA28 in Opisthokonts, showing the high conservation of the150 C-terminal amino acids. (*C*) Phylogenetic relationships of Excavate PA26 relative to PA28 from other supergroups. MrBayes and ML trees were generated from PA28 and PA26 C-terminal sequences MSA. Only PP values above 0.7 are indicated.

**Fig. 6.—:**
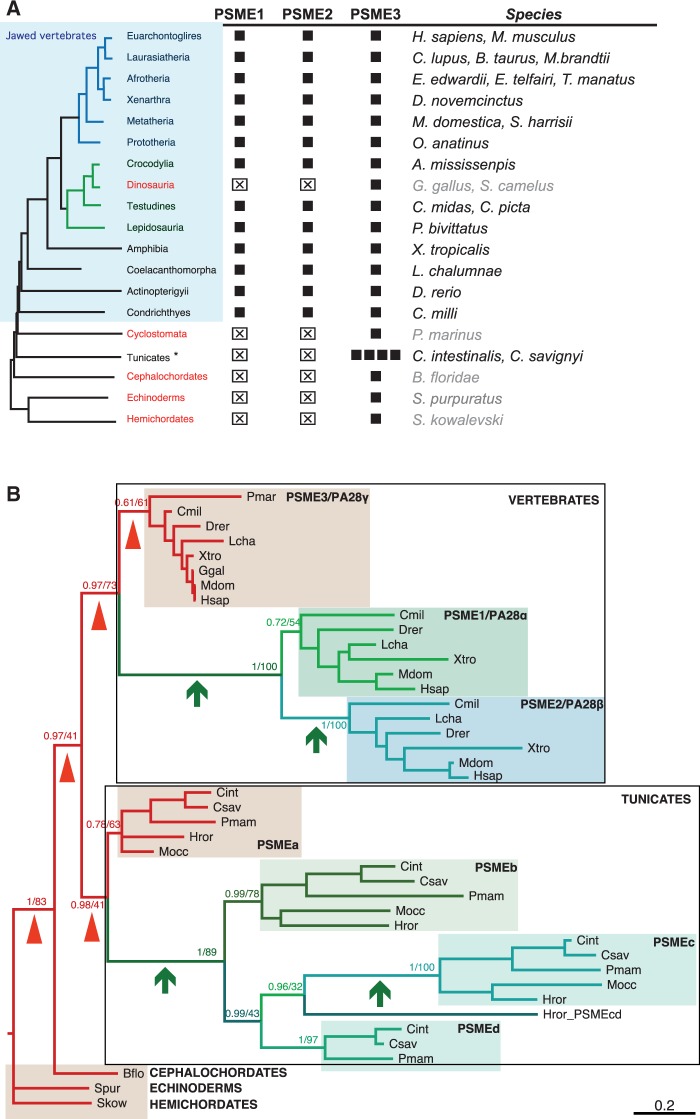
Duplications and losses of PA28 copies in chordates. (*A*) Synopsis of PA28 isoform distribution in chordates. Sequences similar to PSME1 (REGα/PA28α), PSME2 (REGβ/PA28β), and PSME3 (REGγ/PA28γ) were identified by reciprocal Blast searches. Black squares: E-values lower than e^−80^. ⊠: homologous sequences not found. Taxonomy is indicated on the left. Chordate taxa showing a single isoform are colored in red and the names of the corresponding species are grayed. (*B*) PSME phylogeny in chordates. MrBayes and PhyML analysis of the MSA shown in supplementary figure S4, Supplementary Material online, produced the same tree topology. PP and bootstrap proportion are shown only for nodes informative for the duplication history. Short branches that link PSME3 orthologs are signaled by arrowheads and long branches that connect PSME3 to its paralogs, by arrows. Species abbreviation corresponds to species listed in (*A*).

**Fig. 7.—:**
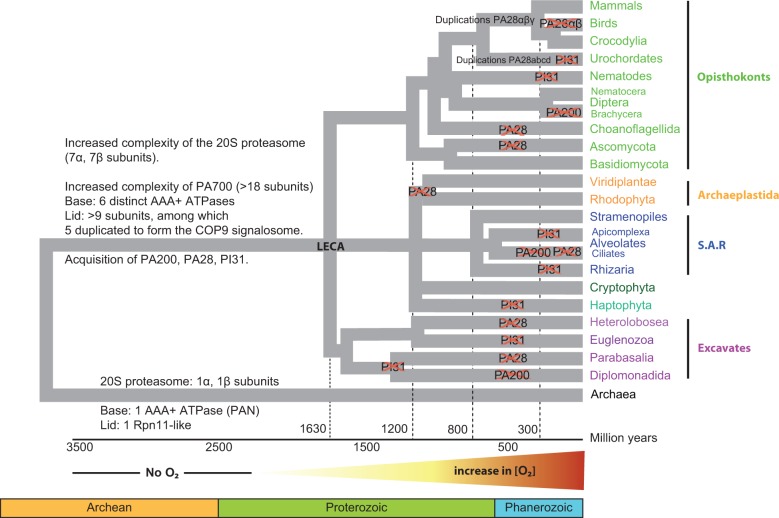
Summary of the distribution of 20S proteasome and proteasome regulators subunits across eukaryote supergroups. The purpose of the timeline of eukaryote emergence, adapted from the Time Tree web page (http://www.timetree.org/index.php, last accessed April 28, 2015), is to give a global view of the supergroups and taxa examined here and of the gain and loss events that have built the current repertoire of proteasome regulators. Note that the complexification process or loss of regulators (crosses) cannot be dated precisely.

MrBayes consensus trees were generated after two independent runs of four Markov chains for 1,100,000 generations sampled every 200 generations, with sampled trees from the first 100,000 generations discarded as burn-in. Average standard deviation of split frequencies were below 0.01 at the end of each run. We also verified that in each case the estimated sample sizes (ESS) were above 200 for all sampled parameters: minimum ESS values were 213.26 (fig. 1*B*), 652.13 (fig. 2*B*), 755.96 (fig. 2*C*), 1145.25 (fig. 3*C*), 2710.73 (fig. 4*E*), 1973.24 (fig. 5*C*), and 1166.98 (fig. 6*B*). Tree samples were summarized by computing a 50% majority-rule consensus tree with associated clade PPs. Trees were visualized and exported as PDF files with FigTree (v1.4.2, http://tree.bio.ed.ac.uk/software/figtree/, last accessed April 28, 2015) then assembled in Adobe Illustrator. Divergence times between taxa presented in figure 7 were collected from the TimeTree database (Hedges et al. 2006) (http://www.timetree.org/index.php, last accessed April 28, 2015).

## Results and Discussion

### PA700 Is Strongly Conserved throughout Eukaryota

The complex formed between the 20S proteasome and the regulatory particle (RP) PA700 (or 19S complex) specifically recognizes and degrades polyubiquitylated substrates. The regulatory role of PA700 is to unfold and deubiquitylate substrates, to both give them access to and to inject them into the 20S proteolytic core. PA700 contains at least 18 core subunits and can break into two subparticles under certain conditions (Glickman et al. 1998); the base, which is composed of the hexameric ATPases PSMC/Rpt 1–6 and the non-ATPase regulatory subunits PSMD1/Rpn2, PSMD2/Rpn1, and ADRM1/Rpn13; the lid, made of PSMD3/Rpn3, PSMD6, 7, 8 (Rpn7, 8, and 12), PSMD11, 12, 13, and 14 (Rpn5, 6, 9 and 11). PSMD14/Rpn11 forms a dimer with PSMD7/Rpn8 and catalyzes deubiquitylation (Verma et al. 2002). Initially thought to bridge the base and the lid because its absence destabilizes their interaction (Glickman et al. 1998), the ubiquitin receptor PSMD4/Rpn10 subunit was later shown to only bind to lid subunits (Tomko and Hochstrasser 2013). Other subunits/proteins, such as Sem1/DSS1/Rpn15 or the deubiquitylating enzymes UCH37 or UBP6, have not been included in this analysis, as they are not always observed in highly purified 26S proteasome samples.

By using a combination of annotation and similarity searches, we identified orthologs of most of the base and lid PA700 proteins in species from all eukaryotic clades examined (fig. 1*A* and supplementary table S1, Supplementary Material online). In particular, we found sequences highly similar to the six AAA+ ATPase subunits. Because eukaryote genomes encode many AAA+ proteins whose ATPase domains share many invariant positions, the finding of proteins similar to PSMC/Rpt ATPases did not necessarily imply that they were true orthologs. We addressed this issue by Bayesian and ML phylogenetic analyses, using the two ATPase domains of Cdc48 as outgroups. In all species examined, the identified six PSMCs, grouped into well-supported clusters along with each vertebrate and yeast PSMC (fig. 1*B*), which gives strong support for their orthology (numbers at nodes indicate PPs [MrBayes] and bootstrap percentages [PhyML]). The relative positions of PSMC groups in figure 1*B* also supports a scenario in which duplications of a unique ancestral PSMC led to the successive emergence of PSMC6, PSMC2, and then PSMC1/4 and PSMC3/5, which duplicated subsequently. This scenario is different from the one proposed from parsimony and neighbor-joining tree analyses of a reduced PSMC sequence set, suggesting a first duplication leading to PSMC2/5/6 and PSMC1/3/4 ancestors (Wollenberg and Swaffield 2001). However, given the importance of the timescale and the relatively low node supports in both studies, there is no definitive argument that may favor either scenario. Recent work showed that archaeal proteasome-activating nucleotides (PAN) and eukaryotic PSMCs associate first as dimers (PSMC1/PSMC2, PSMC3/PSMC6, and PSMC4/PSMC5), which next assemble into a hexamer (reviewed in Tomko and Hochstrasser [2013]). Dimerization of archaeal PANs is mediated by their N-terminal helices, which can adopt *cis* and *trans* conformations thanks to the presence of a proline residue and form a coiled coil of alternating helices in *cis* and *trans* conformations (Djuranovic et al. 2009; Zhang et al. 2009). The proline residue is conserved in eukaryotic PSMC1/3/4 while it is substituted by a lysine in PSMC2 and by a glycine in PSMC5/6. Consequently, PSMC2/5/6 adopt only *trans* conformations and can thus dimerize only with PSMC1/3/4. Because the ancestral PSMC, like PAN, contained a proline, the first duplication event generated two PSMCs with proline residues, one of which conserved the proline whereas the other lost it. The two duplication scenarios share this first sequence of events and are thus both compatible with the currently accepted model for the arrangement of ATPase subunits.

Homologs for the three non-ATPases subunits of the base and the nine subunits of the lid were present in all lineages, except ADRM1/Rpn13, PSMD3/Rpn3, and PSMD8/Rpn12, which were not found in Diplomonads and ADRM1/Rpn13, not found in Parabasalia. These two clades also show a lower conservation of lid subunits, which may reflect a synapomorphic trait because they both belong to Metamonads. In most cases, orthology was not only supported by highly significant BLAST scores (E-value below e^−^^100^) but also by the presence of conserved functional domains: a PH-like domain (Pleckstrin Homology; PF04683) in ADMR1/Rpn13, a PCI domain (Proteasome Cop Initiation factor; PF01399) in PSMD3, 6, 8, 11, 12, 13, a RPN domain (Proteasome Regulatory subunit C-terminal; PF08375) in PSMD3, a JAB/MPN (Mpr1/Pad1 N-terminal; PF01398) in PSMD7/Rpn8 and PSMD14/Rpn11, and a von Willebrand Factor A domain (VWA; PF00092) responsible for the binding of PSMD4/Rpn10 to the ubiquitin-like modifier (UbL) FAT10 and possibly other UbLs (Rani et al. 2012) (supplementary fig. S1, Supplementary Material online). We also excluded the possibility that the identified proteins were actually subunits of the COP9 signalosome (CSN) complex, paralogous to eight subunits of the 26S proteasome lid (Wei et al. 1998). In most eukaryote supergroups, we found the complete set of CSN subunits (supplementary fig. S2 and table S2, Supplementary Material online) and these were distinct from the 26S proteasome lid subunits we identified previously, indicating that the two 26S proteasome and CSN complexes were already present in the LECA. Notable exceptions are Diplomonads, in which we could not identify any CSN subunit, and Parabasalia and Euglenozoa, in which only four and five CSN proteins could be identified, respectively.

These data indicate that PA700 is present in all examined eukaryotic clades. Moreover, in most situations, we found orthologs for the complete subunit set, indicating that the current PA700 structure was acquired before the eukaryotic radiation. Only in the fast evolving *Giardia* and *Trichomonas* (Metamonada, Excavates) did we fail to detect ADRM1/Rpn13, PSMD3/Rpn3, and PSMD8/Rpn12 (fig. 1*A*). The failure to detect these subunits may result from high sequence divergence because other PA700 subunits are globally less conserved in Metamonadida, in agreement with the deep divergence of these protists in the eukaryotic tree (Morrison et al. 2007; Baurain et al. 2010). On the other hand, the missing PA700 subunits may well be true losses, as seems to be the case for the CSN subunits (supplementary fig. S2, Supplementary Material online). Indeed, these protists display peculiar features, for example, they live in anaerobic conditions and lack mitochondria and oxidative phosphorylation enzymes (Muller 1988). For instance, ADMR1/Rpn13 may be dispensable under such particular physiological conditions because it is not critical for basic cell functions as invalidated Rpn13^−/−^ mice developed normally to adulthood (Al-Shami et al. 2010). Such an apparently ancillary role of Rpn13 is in agreement with its location in the distal part of the complex (Lasker et al. 2012).

Whatever the reasons for the sporadic lack of a small number of proteins in particular clades, the overall conservation of PA700 components in all eukaryote supergroups is striking and implies that it was already present in LECA.

### Ancestral PA700 in Archaea

Since nearly all PA700 subunits are encoded by all eukaryotic genomes examined, we next performed extensive TBLASTN, BLASTP, and PHI-BLAST searches in archaea genomes and proteomes to identify which subunits they might encode. Whatever the algorithm used, searches for most subunit sequences in archaea produced hits with low scores (E-values from e^−^^03^ to e^−^^05^) and corresponded to irrelevant proteins. However, PSMC/Rpt AAA+ ATPases and PSMD14/Rpn11 produced hits with much lower E-values (e^−^^68^ to e^−^^72^ and e^−^^14^ and e^−^^18^, respectively), suggesting that they might correspond to orthologs (fig. 2*A* and supplementary table S1, Supplementary Material online). The archaeal PSMC-related ATPases identified produced higher BLAST scores with PSMC than with CDC48 (e^−^^77^ to e^−^^102^ vs. e^−^^41^ to e^−^^58^, supplementary table S3, Supplementary Material online). PSMC-related sequences were unique in each archaea species and highly similar (85–97%) to the archaeal PAN, which was identified in 1996 from the genome of *Methanococcus jannaschii* (Bult et al. 1996) and shown to stimulate activity of the archaeal 20S (Zwickl et al. 1999; Benaroudj and Goldberg 2000). Although more similar to PSMCs among AAA+ATPases (Beyer 1997), the orthology of PAN had not been formally demonstrated. We addressed this issue by using probabilistic phylogenetic approaches (fig. 2*B*). In addition to Cdc48 domains, we also included p60 katanin ATPase domains as outgroups, as these belong to a classical AAA+ subfamily distinct from that of PA700 ATPases (Iyer et al. 2004). The analysis showed that the archaeal PAN sequences are the closest relatives of PSMCs. All PAN sequences branched at the PSMC root, which supports the notion that the six PSMCs were duplicated after the archaea/eukaryote divergence and before the eukaryotic radiation. The presence of a PA700-like structure in archaea is in agreement with the widespread presence of ubiquitin-like proteins and associated conjugating and deconjugating enzymes, and their evolutionary connection with prokaryotic sulfurtansferases (Hochstrasser 2009).

Of the non-ATPase PA700 subunits, only the lid subunit PSMD14/Rpn11 produced two potential orthologs, corresponding to archaeal proteins containing an MPN-like domain (WP_019177758, *Methanomassiliicoccus luminyensis*, Euryarchaeota; Methanomicrobia; YP_008797954, *Candidatus Caldiarchaeum subterraneum*, Thaumarchaeota). This latter species was recently described as a novel archaeal group, encoding a eukaryote-type UbL system made of Ubl, E1, E2, and a small Zn RING finger protein (Nunoura et al. 2011). Although this study mentioned the presence of the YP_008797954 protein, it did not further investigate its relationships with eukaryotic and archaeal MPN-containing proteins.

We thus examined the phylogenetic positions of the WP_019177758 and YP_008797954 proteins with respect to eukaryotic PSMD14/Rpn11 and PSMD7/Rpn8 and to the two groups of archaeal metalloenzymes, which also contain JAB1/MPN/MOV34 (JAMM) domains shown to cleave the ubiquitin-like small archaeal modifier proteins (Hepowit et al. 2012). Phylogenetic analysis of MPN domains showed that the two archaeal PSMD14/Rpn11-related proteins are closer to eukaryotic PSMD7 and PSMD14, and clearly branch outside the two archaeal metalloenzyme groups (fig. 2*C*).

In conclusion, we show that in addition of being functional homologs, the archaeal PANs are true orthologs of eukaryotic PSMCs. Some archaea also encode PSMD14/Rpn11 orthologs, suggesting that these organisms might express a PA700-like complex made of a simple base and lid structure, although further biochemical confirmation will be required to confirm this.

Although the split between archaea and eukaryotes occurred long before LECA, from 1.5 to 2.5 billion years depending on the methods used (Eme et al. 2014), this constitutes a puzzling evolutionary issue as to how the RP has adapted from a simple two-component structure in archaea (the homohexameric PAN and the Rpn8/Rpn11 ortholog in some species) to a 18-component complex in LECA. The same is true for the 20S structure, which consists of at least 14 different monophyletic subunits (7 α and 7 β) in eukaryote supergroups, whereas most of archaea encode unique α and β proteins (Bouzat et al. 2000). The situation is even more puzzling knowing that the binding of the ATPase hexamer to the α ring adds to the selective constraints.

### PI31 Is Present throughout Eukaryota but Was Lost in Multiple Lineages

PI31 (proteasome inhibitor 31 kDa, PSMF1) is the least studied proteasome regulator. Originally identified as an in vitro 20S proteasome inhibitor (Chu-Ping et al. 1992), orthologs were identified in the genomes of various metazoans and yeast (Botelho-Machado et al. 2010). Although the exact role of PI31 in cells is still a matter of debate (Li et al. 2014), recent studies indicate that it can activate the 26S proteasome in vitro and positively control proteasome activity in living cells; in *Drosophila*, loss of PI31 function is lethal, indicating a basic cell function. PI31 is also involved in sperm differentiation by controlling proteasome activity and this requires interaction with the F-box only Nutcracker protein (Bader et al. 2011). In the yeast, the PI31 homolog Fub1p is involved in the control of boundaries between transcriptionally active and inactive chromatin domains (Hatanaka et al. 2011). Fub1p interacts physically with multiple 20S proteasome α and β subunits and genetically with its 19S regulatory complex. Loss of Fub1p function produced no phenotype (Yashiroda et al. 2015) but showed synthetic lethality in interaction with the loss of the Pba3 proteasome chaperone.

We searched for PI31-like sequences in eukaryotic supergroups (fig. 3 and supplementary table S4, Supplementary Material online). We identified PI31 sequences in most Opisthokonts excluding three tunicate species and nine nematode species, suggesting that it was lost in these two clades. We detected PI31-related sequences in Amoebozoa, Archaeplastida (green plants and red algae), and Cryptophyta but could not find any PI31-related sequences in Apusozoans and in Haptophyta. The situation was less straighforward in other supergroups becausewe detected sequences distantly related to PI31 in a limited number of clades; in Excavates, it was identified only in Heterolobosea, in Stramenopiles, only in *Blastocystis* and Oomycetes, and in Alveolates, only in Ciliophora. Sequence similarity in these clades was mostly restricted to the C-terminal proline-rich motif, which mediates dimerization with the ubiquitin-ligase Fbxo7 (Kirk et al. 2008). Although PI31 and Fbxo7 showed sequence similarity in their C-termini (fig. 3*B*), the sequences we identified were bona fide PI31 orthologs as demonstrated by phylogenetic analysis (fig. 3*C*).

Our survey of PI31 orthologs showed that it is poorly conserved across eukaryotic supergroups and was most likely lost in several clades, like tunicates and nematodes within opisthokhonts. It was also probably lost in other supergroups in which we could not detect it, although the general lack of sequence conservation for this proteasome regulator does not allow the drawing of definitive conclusions. The presence of PI31 homologs in at least one clade in Excavates and in SAR indicates that this regulator was nevertheless likely present in LECA. However, given our limited knowledge of its physiological role, there is no firm biological ground to interpret the observed pattern of presence and absence in eukaryotes.

### PA200 Was Present in LECA but Was Lost in Specific Lineages

PA200 was first identified in rabbit reticulocyte lysates (Hoffman et al. 1992) and further found in nematodes, yeast (known as Blm10p), and plants (Ustrell et al. 2002). Yeast lacking Blm10p are hypersensitive to DNA-damaging agents and showed reduced respiratory capacity (Sadre-Bazzaz et al. 2010; Doherty et al. 2012). However, PA200 knockout mice did not show higher sensitivity to DNA-damaging agents but displayed a severe reduction in male fertility (Khor et al. 2006), resulting from the failure to degrade acetylated core histones in elongated spermatids (Qian et al. 2013).

PA200/Blm10 is a large monomeric protein containing numerous ARM/HEAT repeats, which confer α-helical solenoid structures globally arranged into a dome with a 13–22 Å aperture in its center (Kajava et al. 2004; Sadre-Bazzaz et al. 2010). PA200 binds to the 20S α-rings and thus induces conformational changes in the gate (Schmidt et al. 2005; Dange et al. 2011). There is currently some controversy as to whether PA200 can or cannot facilitate the entrance of substrates into the 20S proteasome (Ortega et al. 2005; Schmidt et al. 2005; Iwanczyk et al. 2006; Sadre-Bazzaz et al. 2010; Dange et al. 2011).

Despite the fact that the PA200 3D structure appears well conserved in vertebrates and yeast (Kajava et al. 2004), its amino acid sequence is only moderately conserved (17% identity and 38% similarity between human and yeast (Ustrell et al. 2002)). The C-terminus shows higher conservation (34% identity and 50% similarity). This corresponds to a 100–130 amino acid domain referred to as the Pfam domain PF11919, known to bind in pockets formed by the 20S α5 and α6 subunits and to induce 20S gate opening (Ortega et al. 2005; Sadre-Bazzaz et al. 2010). We thus examined PA200 distribution across eukaryotic supergroups, by using either full-length sequences or the Pfam PF11919 domain as queries for BLAST searches.

We identified proteins similar to PA200 in all eukaryotic supergroups examined (Opisthokonts, Amoebozoans, Apusozoans, Excavates, Archaeplastida, SAR, Cryptophyta, and Haptophyta; fig. 4*A*). The PA200-like proteins identified were coded by unique genes in each genome and all harbored the PF11919 domain at their C-termini. This domain was itself found only once in each genome and can thus be considered as a signature for PA200 orthology. In contrast, Archaea genomes did not contain any PF11919 domain nor did they encode protein related to PA200, even distantly.

Apart from their C-termini, the proteins identified in the eukaryotic supergroups did not have sequence motifs in common; therefore we applied the profile approach (Kajava et al. 2004), which detected a number of HEAT-like repeats dispersed along the Blm10/PA200 proteins. This suggested that the proteins identified have α-solenoid folded structures similar to the known crystal structure from *S*acch*. cerevisiae* (fig. 4*B*, Sadre-Bazzaz et al. 2010). Structure comparison of these proteins with that of *S*acch*. cerevisiae* Blm10 (fig. 4*C*) confirmed likelihood of the similar α-solenoidal structure, with several putative insertions specific for each protein. Analysis of the crystal structure of the *S*acch*. cerevisiae* Blm10 revealed that insertions of sizes longer than 40 residues might protrude without affecting the core of the α-solenoid structure (fig. 4*D*). Thus, despite the low sequence similarity between Blm10/PA200 sequences, one may conclude that these proteins display similar overall 3D structures, only differing from each other by a few insertions of different sizes and locations.

While analyzing BLAST searches results, we noticed that PA200 was not found in the genomes of several taxa: in Alveolates, we only found PA200 in Apicomplexa (supplementary table S5, Supplementary Material online) and could not detect it in Ciliophora (eight genomes, among which *Paramecium tetraurelia* and *Tetrahymena thermophila*), in Chromerida (*Chromera velia*), and in Perkinsea (*Perkinsus marinus*). Alveolates are currently considered to comprise two clades: The Ciliate clade and a clade made of the two monophyletic lineages Apicomplexa and Dinoflagellates (Bachvaroff et al. 2011). The absence of PA200 in Ciliates and Dinoflagellates therefore suggests that the gene was lost at least twice, once in each clade. Another possibility, although less likely, is that PA200 may have originally been absent in Alveolates and later acquired in Apicomplexa by horizontal gene transfer. A precedent for this has already been established for other genes (Templeton et al. 2004; Balaji et al. 2005; Kishore et al. 2013).

We also failed to detect PA200 in *Giardia* (Diplomonads), for which six genomes are available (Morrison et al. 2007; Jerlstrom-Hultqvist et al. 2010). Absence of PA200 in *Giardia* may result either from too high a divergence or from a true loss due to the particular metabolism of this species. We favor the latter scenario since we detected PA200 in other highly divergent excavate species (i.e., *T. vaginalis*, *L. major* or *N. gruberi*). More puzzling is the absence of PA200 in the genomes of Brachycera dipterans (flies), whereas it was readily identified in other insect clades (dipteran Nematocera [six species], Hymenoptera [ten species], Coleoptera [one species], Lepidoptera [two species], and Paraneoptera [one species]), crustaceans (Branchiopoda and Copepoda) and chelicerates (two arachnid species; supplementary table S5, Supplementary Material online). We could not find PA200 homologs in the genomes of 17 Brachycera species from six different superfamilies (12 *Drosophila* species, *Ceratitis capitata*, *Musca domestica*, *Glossina morsitans*, *M. abdita,* and *E. balteatus*; supplementary table S5, Supplementary Material online). Comparison of the Pfam PF11919 domains found in other arthropods revealed a high level of similarity (fig. 4*E*), which makes it unlikely that PA200 orthologs exist in Brachycera but are too divergent to be detected. The finding that specific insects have lost PA200 raises interesting questions about what specific aspects of their physiology correspond to a bypassing of the functions normally controlled by PA200 in other species. PA200 has been proposed to play roles in mitochondria fission (Tar et al. 2014) and DNA repair (Ustrell et al. 2002). Deficiency in these two processes probably accounts for the spermatogenesis defects observed in PA200^−/−^ mice (Khor et al. 2006; Qian et al. 2013). One possibility is that Brachycera have replaced PA200 activity in spermatogenesis by other proteasome-dependent pathways, perhaps through ubiquitylation (Bader et al. 2011). Alternatively, adaptive selection of other DNA repair or mitochondria metabolic pathways in Brachycera might have superseded the processes normally controlled by PA200. One example of protein with such potential features could be the ribosomal subunit RpS3, whose amino acid sequence in Brachycera contains a critical Q59 glutamine residue that confers it with extraribosomal lyase and *N*-glycosylase activities involved in DNA repair (Wilson et al. 1993; Deutsch et al. 1997). The Q59 residue is not present in RpS3 of other insects, including Nematocera (Li and Fallon 2006), or of metazoans, fungi, and plants (Lyamouri et al. 2002). Interestingly, the *Drosophila* RpS3 protein is also involved in Reactive Oxygen Species (ROS)-mediated mitochondrial DNA repair (Kim et al. 2013), as is the case for PA200 (Sadre-Bazzaz et al. 2010; Tar et al. 2014).

In conclusion, the identification of PA200-like proteins in all eukaryotic groups examined indicates that this activator was present in LECA. However, PA200 was lost in several taxa within the Alveolates, Metamonadida, and Diptera, indicating that this protein fulfills specific physiological functions that became dispensable in particular life conditions.

### PA28 Was Present in LECA but Was Lost in Several Eukaryotic Supergroups

PA28, also known as PSME, REG, or 11S, was first identified in bovine red blood cells and heart (Ma et al. 1992). PA28 assembles as heptameric rings that cover the top of the 20S cylindrical chamber and is anchored into pockets between α subunits. PA28 homologs have been identified in arthropods (*Drosophila melanogaster* and *Ixodes scapularis*; Masson et al. 2001) and Platyhelminthes (Schistosoma; Soares et al. 2013), and more distantly related proteins are also known in protists such as Amoebozoa (*Dictyostelium discoideum*; Masson et al. 2009) and Euglenozoa (PA26 in *Trypanosoma brucei*; Yao et al. 1999). PA28 is involved in the control of cell cycle and apoptosis (Murata et al. 1999; Masson et al. 2003) by facilitating the degradation of the cyclin-dependent kinase inhibitor p21 and p53 (Chen et al. 2007; Li et al. 2007; Zhang and Zhang 2008). In keeping with these basic functions, knockout mice for PA28γ showed reduced body size and cell-specific mitosis defects (Murata et al. 1999). A PA28 ortholog also exists in *Caenorhabditis elegans* (Y66D12A.9), which was shown to physically interact with ccm-3, itself homolog to the vertebrate programmed cell death protein 10 (Li et al. 2004). RNAi-mediated PA28 knockdown had no apparent effect on the development and morphology of wild-type worms but it suppressed the Daf-c phenotype elicited in the *p673* mutant of the *daf-21* gene encoding Hsp90 (Minami et al. 2006).

We examined the presence of PA28 in the same panel of eukaryotic supergroups as used above for PA700, PI31, and PA200. We identified single-copy PA28 sequences in the genomes of species from many supergroups (Opisthokonts, Amoebozoans, SAR, Cryptophyta, and Haptophyta). However, PA28-like sequences were absent in several supergroups and specific lineages (fig. 5*A* and supplementary table S6, Supplementary Material online). In Opisthokonts, PA28 is absent in Choanoflagellates (two genomes) and in Ascomycota (>100 genomes), whereas it is present in Basidiomycota. Since PA28 sequences in Opisthokonts share above 30% identity over the last 150 amino acids (fig. 5*B*), the perceived absence of PA28-like sequences in Choanoflagellates and Ascomycota can thus be most likely considered indicative of genuine losses.

In the three clades of the SAR kingdom, we identified sequences that shared 18–45% identity with human PA28γ. However, no PA28-like sequence could be detected in Ciliophora and Chromerida (Alveolates), whereas it was present in *Reticulosa filosa* (Rhizaria). PA28 was also absent from a major kingdom, Archaeplastida; we could not detect any sequence significantly related to PA28, either in green plants (9 green algae and 111 angiosperm genomes available) or in red algae (4 genomes available).

Excavates showed a more complex situation since Euglenozoa express a functional homolog of PA28 (PA26, *T. brucei*), which is highly divergent in its primary sequence (To and Wang 1997; Yao et al. 1999). In agreement, BLAST searches in Excavates using various PA28 sequences as queries identified PA26 with scores commonly considered as nonsignificant (E-values from 1.3 to 8.7). However, it identified the EET00138 protein in *Giardia intestinalis* (Diplomonads) with a more significant score (E-value = 6.44e^−^^08^). Conversely, BLAST searches using EET00138 as query detected metazoan PA28 sequences with better scores than using PA26 (4.05e^−^^03^ to 3.7e^−^^05^ vs. 3.17 to 8.01). This suggests that EET00138 is a PA distantly related to PA28 (supplementary table S7, Supplementary Material online). Phylogenetic analysis (fig. 5*C*) gave further support to this hypothesis, although the lack of outgroup prevents to draw definitive conclusions.

In conclusion, our data indicate that PA28 was likely present in LECA and has been lost secondarily in specific taxa, that is, Ascomycota (yeast) within Fungi or Archaeplastida (green plants and algae). The presence in *Giardia* of a distantly related counterpart of the trypanosome PA26 lends additional support for the common origin of PA28 and PA26. The wide distribution of PA28-like sequences in eukaryotes is consistent with a role in the repair or degradation of damaged proteins. However, PA28 function has been examined only in metazoan models (mice, drosophilids, and nematodes) and it would be highly informative to study its function in unicellular organisms such as *D. discoideum*, in which PA28 gene disruption unfortunately did not produced recombinant amoeba (Masson et al. 2009).

### PA28 Duplicated in Chordates and Specific Copies Were Lost in Birds

Mammals express three PA28-like sequences; PA28α/REGα/PSME1 and PA28β/REGβ/PSME2 (Mott et al. 1994), which assemble as heteroheptamers (Johnston et al. 1997; Zhang et al. 1999), and PA28/REGγ/PSME3, first described as Ki nuclear antigen, which assembles as homoheptamers. Previous studies based on distance methods showed that the unique PA28 of invertebrates was more closely related to the vertebrate PA28γ and placed PA28α and β at positions inconsistent with the accepted species phylogeny (Murray et al. 2000; Masson et al. 2001). However, the genomic data available at the time were sparse, therefore we used the current range of PA28 sequences as well as more accurate phylogenetic reconstruction methods to revisit the evolution of the three PA28 subunits.

We first examined which species are expressing the three PA28 isoforms. As shown in figure 6*A*, we identified the three PA28 isoforms in all jawed vertebrates except birds and platypus. We could only find two annotated PSME genes, PSME2 and PSME3 in the platypus genome assembly. However, we identified four PSME1 specific exons in raw sequence data (supplementary table S8, Supplementary Material online), indicating that the three genes are present in platypus. As of birds, we readily found PSME3 homologs, but could not identify PSME1 and PSME2 sequences in the genomes of 48 species (46 from neognath and two from palaeognath orders, supplementary table S8, Supplementary Material online). In contrast, we found the three PSME genes in Crocodylia (*A. mississipi*), the closest relative of birds. This strongly supports the conclusion that PSME1 and PSME2 genes were both lost in the bird ancestral lineage.

Although we found the three PA28 genes in jawed vertebrates, we detected only a single PA28/PSME in the genomes of the jawless vertebrate lamprey (*P. marinus*; Hyperoartia), the lancelet (*B. floridae*; Cephalochordates), sea urchin (*S*t*. purpuratus*; Echinoderms), and the acorn worm (*S*a*. kowalevskii*; Hemichordates). This supports a scenario according to which two sequential PA28 duplications took place in jawed vertebrates before their radiation between bony and cartilaginous clades.

Interestingly, PSME duplications also occurred in tunicates, the sister group to vertebrates (Delsuc et al. 2006); We detected four PSME genes in the genomes of three species of the Phlebobranchia order (*Ciona intestinalis*, *Ciona savignyi*, and *Phallusia mammillata*) and two species of the Stolidobranchia order (*Halocynthia roretzi* and *Molgula occulata*). This indicates that the duplications took place before the divergence of the two orders more than 350 Ma (Delsuc F, unpublished data). The four tunicate genes encode divergent proteins (31% identity on average, supplementary table S9 and fig. S3, Supplementary Material online) but show much higher conservation across the five species (54–61% identity), which implies that the four genes are functional and evolve under selective constraints. We next addressed whether the three genes in vertebrates and the four genes in tunicates originated from distinct duplication events in each taxon or from a common duplication event that occurred before the divergence between vertebrates and tunicates. Bayesian and ML phylogenetic analyses both inferred the same tree topology, consistent with the currently accepted deuterostome phylogeny (fig. 6*B*). The most salient features are that PSME duplicated independently in tunicates and vertebrates. In these two lineages, PSME3 orthologs appear as slow-evolving sequences (densely packed clusters, in red), from which much faster evolving sequences stemmed out, leading to PSME1/2 (in vertebrates) and PSMEb/c/d (in tunicates). Branches that connect PSME1/2 in vertebrates or PSMEb/c/d in tunicates (arrows) are much longer than those connecting PSME3 across taxa (arrowheads). Thus, the same scenario has occurred in parallel in vertebrates and tunicates: a single paralog, namely PSME3/PA28γ in vertebrates and PSMEa in tunicates, remained highly similar to the unique PSMEs found in other deuterostomes, whereas the other paralogs are all connected by a long ancestral branch, indicating that they originated from a copy that diverged at a high rate soon after the first duplication event (fig. 6*B*). The most likely explanation is that the most conserved paralogs have retained the ancestral PSME function while the others now fulfill new functions associated with adaptive amino acid changes. Such a high and transient evolutionary rate agrees well with studies showing that positive selection and neofunctionalization are major drivers for the retention of duplicate copies in genomes (Shiu et al. 2006; Pegueroles et al. 2013).

Although data concerning the role of PSME in tunicates are sparse, neofunctionalization is well documented in vertebrates as PSME1/PA28α and PSME2/PA28β differ from PSME3/PA28γ in several respects: PA28α and PA28β are cytoplasmic and can form heteroheptamers whereas PA28γ is nuclear and only forms homoheptamers (Tanahashi et al. 1997); PA28α and PA28β are both encoded by interferon gamma (IFNγ)-inducible genes and expressed at high levels in cells specialized in antigen presentation (Macagno et al. 1999). PA28α and PA28β are thought to facilitate the production of antigenic peptides bound by major histocompatibility complex (MHC) class I proteins (Groettrup et al. 1996).

The role of PA28 α and β subunits in the MHC class I presentation pathway has two main implications in terms of evolution; it favors the scenario according to which the original duplication took place between jawless and jawed vertebrates, because the former lacks the IFNγ-inducible MHC components (Kandil et al. 1996) and even probably MHC (Uinuk-ool et al. 2003); it also provides an explanation for the absence of PA28α and PA28β in birds. Indeed, we found that birds also lack LMP2 (β1i), LMP7 (β5i), and MECL-1 (β2i), the three IFNγ-inducible β subunits incorporated into the immunoproteasome (Griffin et al. 1998) (supplementary fig. S4, Supplementary Material online), whereas they were readily identified in Crocodylia (the sister group of Dinosauria/Birds) and the other sauropsids Testudines (turtles) and Lepidosauria (lizards) (Chiari et al. 2012; supplementary table S10, Supplementary Material online). These IFNγ-induced components render immunoproteasomes more efficient in the processing of antigenic viral peptides, as illustrated by alterations of the cytotoxic T lymphocyte repertoires displayed by LMP2, LMP7, and PA28αβ knockout mice (Murata et al. 1999; Chen et al. 2001; Toes et al. 2001). The loss of PA28α and PA28β in birds is thus likely associated with a global loss of all IFNγ-inducible proteasome components and supports the notion that PA28αβ does not significantly participate in physiological functions other than those linked to the immunoproteasome.

## Conclusion

Our study shows that the currently known proteasome regulators are widely expressed in eukaryote supergroups, and establish that they were all already present in LECA. This was expected for PA700, for which a simplified version had already been identified in Archaea. Less expected was the extremely high conservation of PA700 multisubunit complex structure in all eukaryotes. In particular, whereas Archaea express a single AAA+ ATPase base unit, all eukaryotic species examined encode six ATPases, never less. The presence of PA200, PA28, and PI31 in all eukaryotic supergroups gives additional support to the notion that these regulators fulfill basic functions in cell physiology, like DNA repair, control of cell cycle, and apoptosis. Given the paramount importance of these physiological functions in terms of adaptive fitness, the selective losses of PA200 in Brachycera insects and of PA28 in Ascomycota fungi were therefore unexpected and raises the issue as to whether yeast and *Drosophila* are suitable model organisms to address the functions of mammalian PA28 or PA200 because these organisms have a particular physiology adapted to the absence of one PA. Extreme situations are Ciliates (*Paramecium* or *Tetrahymena*), which lack PA200 and PA28, and Diplomonads (*Giardia*), which lack PI31, PA200, all CSN subunits, perhaps a few PA700 subunits, and only express a distantly related thus putative PA26 sequence (fig. 7). Thus, despite the very ancient origin of all proteasome regulators in eukaryotes, the “proteasome toolbox” appears as a dynamic adaptive machinery, whose requirement in eukaryotic cell physiology has greatly varied depending on species biology.

## Supplementary Material

Supplementary tables S1–S10 and figures S1–S3 are available at *Genome Biology and Evolution* online (http://www.gbe.oxfordjournals.org/).

Supplementary Data
