# Supplementary material for: Evolution of Proteasome Regulators in Eukaryotes
Source: Genome Biol Evol. 2015 May 4;7(5):1363–79. doi: 10.1093/gbe/evv068 (PMC4453063; doi:10.1093/gbe/evv068)
Supplement: Supplementary Data [file supp_7_5_1363__index.html]

Evolution of proteasome regulators in Eukaryotes. — Evolution of Proteasome Regulators in Eukaryotes — Supplementary Data 

# Evolution of Proteasome Regulators in Eukaryotes

## Supplementary Data

files

**Files in this Data Supplement:**

- Supplementary Data - pdf file
- Supplementary Data - docx file
- Supplementary Data - xls file
