# Supplementary material for: Evolution of Proteasome Regulators in Eukaryotes
Source: Genome Biol Evol. 2015 May 4;7(5):1363–79. doi: 10.1093/gbe/evv068 (PMC4453063; doi:10.1093/gbe/evv068)
Supplement: Supplementary Data [file supp_evv068_FigureS1-S4.pdf]

Figure S1:Conserved domain organization of non-ATPase PA700 subunits.
Putative protein sequences were annotated by Interproscan (Jones, et al. 2014). PH-like: similar to Pleckstrin Homology (PF04683), VWA: Von Willebrand type A (PF00092), MPN: Mpr1/Pad1 N-terminal (PF01398), PCI: Proteasome Cop Initiation factor (PF01399), PRC: Proteasome Regulatory subunit C-terminal (PF08375).
Alveol: Alveolates, Amoeb: Amoebozoa, Choano: Choanoflagellida, Crypto: Cryptophyta, Euglen: Euglenozoa, Hapto: Haptophyceae, Heterol: Heterolobosea, Ichthyo: Ichthyosporea, Mamm: Mammalia, Rhodo: Rhodophyta, Strameno: Stramenopiles, Viridi: Viridiplantae.

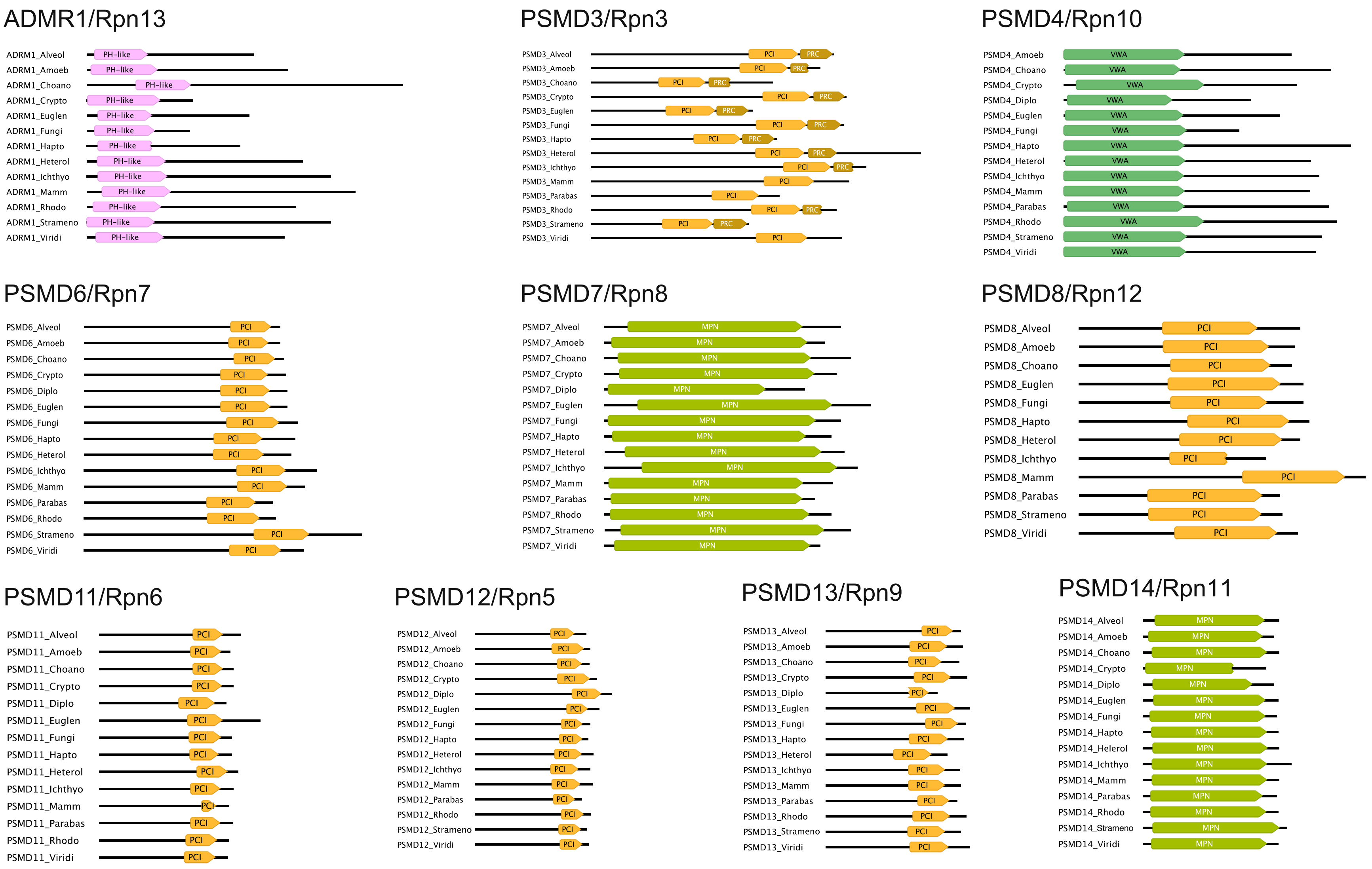

Figure S2: The eight subunits of the COP9 signalosome (CSN) complex are present in most eukaryote supergroups.

	GPS1<br>Csn1	SGN2<br>Csn2	SGN3<br>Csn3	SGN4<br>Csn4	JAB1<br>Csn5	MOV34-34kd<br>Csn6	COPS7a<br>Csn7a	COPS7b<br>Csn7b	HCOP9<br>Csn8	*Species*
**Opisthokonta**										
Metazoa	■	■	■	■	■	■	■	■	■	*H. sapiens*
Choanoflagellida	■	■	■	■	■	■	■		■	*M. brevicollis, S. rosetta*
Ichthyosporea	■	■	■	■	■	■	■		■	*C. owczarzaki*
Fungi	■	■	■	■	■	■	■		■	*P. omphalodes, S. pombe*
**Amoebozoa**	■	■	■	■	■	■	■		■	*D. discoideum, E. histolytica*
**Apusomonads**	■	■	■	■	■	■	■		■	*T. trahens*
**Excavates**										
Discoba										
Heterolobosea	■	■	■	■	■	■	■		■	*N. gruberi*
Euglenozoa	☒	■	☒	■	■	▪	■		☒	*L. major, T. brucei*
Metamonada										
Diplomonadida	☒	☒	☒	☒	☒	☒	☒		☒	*G. intestinalis, G. lamblia, S. salmonicida*
Parabasalia	■	☒	☒	▪	■	☒	▪		☒	*T. vaginalis*
**Archaeplastida**										
Viridiplantae	■	■	■	■	■	■	■		■	*A. thaliana, P. trichocarpa*
Rhodophyta	■	■	■	■	■	■	■		■	*G. sulphuraria*
**SAR**										
Stramenopiles	■	■	■	■	■	■	■		■	*T. pseudonana, P. infestans, N. gaditana*
Alveolates	■	■	■	■	■	■	■		■	*T. thermophila, O. trifallax, S. lemnae*
Rhizaria	■	■	■	■	■	■	■		■	*R. filosa*
**Cryptophyta**	■	■	■	■	■	■	■		■	*G. theta*
**Haptophyceae**	■	■	■	■	■	■	■		☒	*E. huxleyi*

Sequences of the eight CSN subunits from different species were used as Blast queries. Best hits were blasted against nr database. Accession numbers are listed in Table S2.

☒ No protein found
■ E-value lower than e^{-60}
▪ E-value between e^{-5} and e^{-15}

Figure S3: Multiple alignement of deuterostome PA28 sequences.

Deuterostome sequences identified in TableS8 were aligned with Mafft. Conserved residues are in colored background. Hsap: Human, Mdom: Opossum, Ggal: Chicken, Xtro: Xenopus, Lcha: Cœlacanth, Drer: zebrafish, C.mil: Elephant shark, Pmar: Lamprey, Bflo: Lancelet, Spur: Sea urchin, Skow: Acorn worm, Cint: Ciona intestinalis, Csav: Ciona savignyi, Pmam: Phallusia mammillata, Hror: Halocynthia roretzi, Mocc: Molgula occulta.

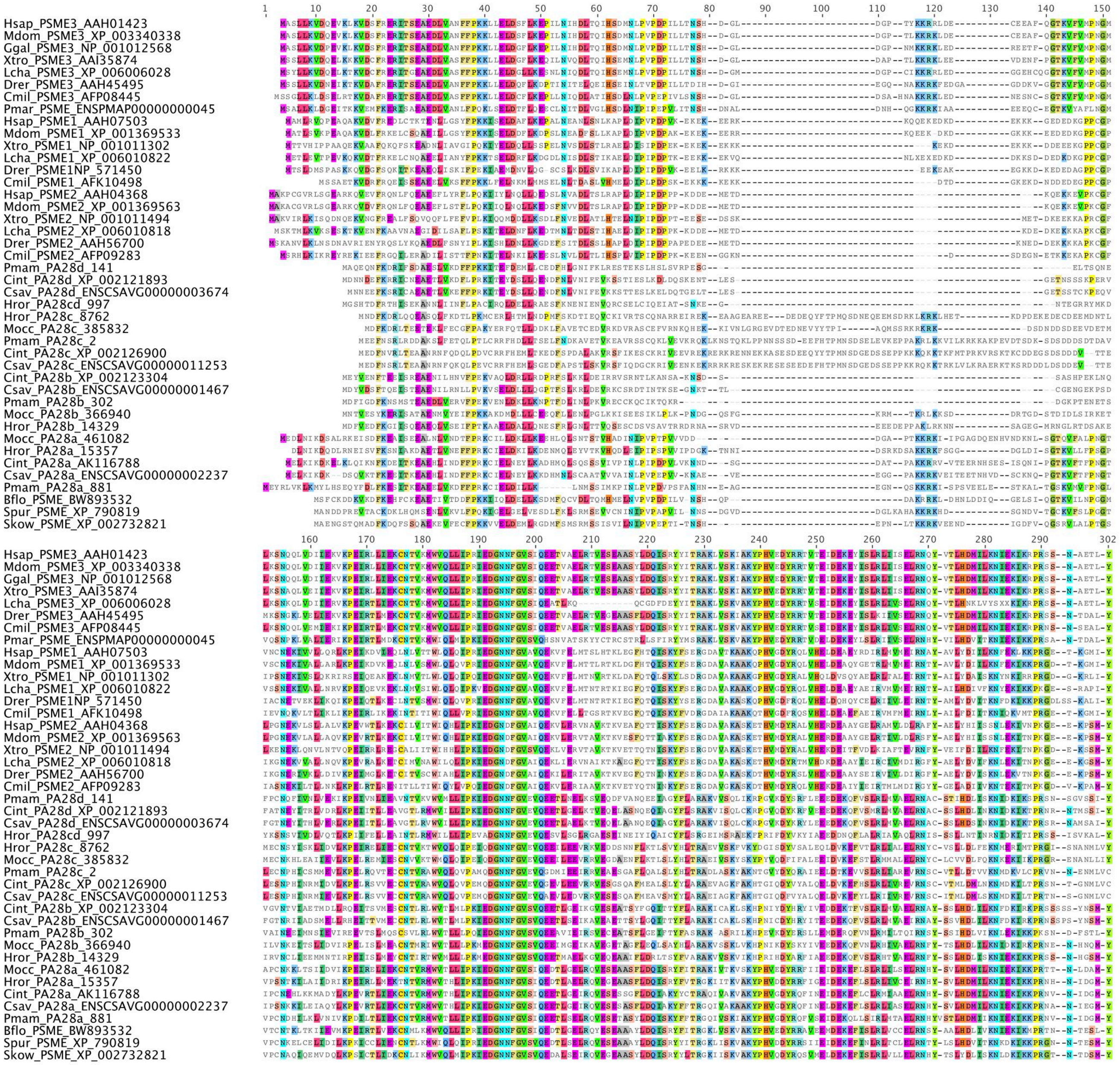

Figure S4: IFNγ-inducible β subunits are absent in birds.

Clade	PSMB6 LMP-Y(β1)	PSMB9 LMP2(β1i)	PSMB-7 LMP-Z (β2)	PSMB10 MECL-1(β2i)	PSMB5 LMP-X(β5)	PSMB8 LMP7(β5i)	Species
Sarcopterygii / Tetrapodia / Amniota / Synapsida (mammalia)	■	■	■	■	■	■	*H. sapiens*
Sauropsida / Lepidosauria	■	■	■	☒	■	■	*A. carolinensis*
Archosauria + Testudines / Archosauria / Crocodyla	■	■	■	☒	■	■	*A. mississipiiensis*
Archosauria + Testudines / Archosauria / Dinosauria	■	☒	■	☒	■	☒	*G. gallus*[a]
Archosauria + Testudines / Archosauria / Dinosauria	■	☒	■	☒	■	☒	*M. gallopavo*[a]
Archosauria + Testudines / Archosauria / Dinosauria	■	☒	■	☒	■	☒	*T. guttata*[b]
Archosauria + Testudines / Archosauria / Dinosauria	■	☒	■	☒	☒	☒	*C. brachyrhynchos*[b]
Archosauria + Testudines / Testudines	■	■	■	☒	■	■	*C. picta bellii*
Amphibia	■	■	■	☒	■	■	*X. tropicalis*
Dipnoi (lunfishes)	■	■	■	■[c]	■	■	*P. dolloi*[d]
Actinopterygii (ray-finned fishes)	■	■	■	■[c]	■	■	*D. rerio*

(LMP spans the column headings PSMB6 to PSMB8.)

[a] Belong to the Galliformes order.

[b] Belong to the Passeriformes order.

[c] The presence of PSMB10 in fishes/lungfishes and in mammals implies that it was lost at least twice, in sauropsids and amphibians.

[d] Tacchi et al. 2013. Dev. Comp. Immunol. **41**:657-665.

☒ No protein found

■ E-value lower than e^{-60}
