# Supplementary material for: Evolution of Proteasome Regulators in Eukaryotes
Source: Genome Biol Evol. 2015 May 4;7(5):1363–79. doi: 10.1093/gbe/evv068 (PMC4453063; doi:10.1093/gbe/evv068)
Supplement: Supplementary Data [file supp_evv068_New_Microsoft_Office_Word_Document.docx]

# Supporting Information

Table S1: Accession numbers for orthologs of the 18 PA700 subunits in eukaryote supergroups.

Table S2: Accession numbers for orthologs of the 8 COP9 signalosome subunits.

Table S3: Accession numbers and E-values for the archaeal orthologs of eukaryotic PA700 subunits.

Table S4: Accession numbers for PI31 orthologs in eukaryotes.

Table S5: Accession numbers for PA200 orthologs in eukaryotes.

Table S6: Accession numbers for PA28 orthologs in eukaryotes.

Table S7: Accession numbers and E-values for PA28/PA26 orthologs in Excavata.

Table S8: Accession numbers for PA28 isoforms in vertebrates.

Table S9: Amino acid identity matrix of PSME proteins in deuterostomes.

Table S10: Accession numbers for IFNγ‑inducible LMPs in Sauropsida.

Figure S1: Conserved domain organization of non-ATPase PA700 subunits.

Figure S2: Distribution of COP9 signalosome components in eukaryote supergroups.

Figure S3: Multiple alignment of deuterostome PA28 sequences

Figure S4: Specific loss of IFNγ‑-inducible β 20S subunits in birds.
